# Patterns of postural sway in high anxious children

**DOI:** 10.1186/1744-9081-5-42

**Published:** 2009-10-02

**Authors:** John F Stins, Annick Ledebt, Claudia Emck, Elisabeth H van Dokkum, Peter J Beek

**Affiliations:** 1 Research Institute MOVE, Faculty of Human Movement Sciences, VU University Amsterdam, van der Boechorststraat 9, 1081 BT, Amsterdam, The Netherlands

## Abstract

**Background:**

Current research suggests that elevated levels of anxiety have a negative impact on the regulation of balance. However, most studies to date examined only global balance performance, with little attention to the way body posture is organized in space and time. The aim of this study is to examine whether posturographic measures can reveal (sub)clinical balance deficits in children with high levels of anxiety.

**Methods:**

We examined the spatio-temporal structure of the centre-of-pressure (COP) fluctuations in children with elevated levels of anxiety and a group of typically developing children while maintaining quiet stance on a force plate in various balance challenging conditions. Balance was challenged by adopting sensory manipulations (standing with eyes closed and/or standing on a foam surface) and using a cognitive manipulation (dual-tasking).

**Results:**

Across groups, postural performance was strongly influenced by the sensory manipulations, and hardly by the cognitive manipulation. We also found that children with anxiety had overall more postural sway, and that their postural sway was overall less complex than sway of typically developing children. The postural differences between groups were present even in the simple baseline condition, and the group differences became larger with increasing task difficulty.

**Conclusion:**

The pattern of postural sway suggests that balance is overall less stable and more attention demanding in children with anxiety than typically developing children. The findings provide further evidence for a neuro-behavioral link between psychopathology and the effectiveness of postural control.

## Background

The control of quiet upright stance is accomplished through a delicately orchestrated activation of the musculoskeletal system, which involves a combination of vestibular, visual, and somato-sensory inputs (see [1]). These inputs are part of neural feedback mechanisms that operate through, and along, the spinal cord and the brainstem for the purpose of balance control [2]. Furthermore, various higher brain structures like basal ganglia, cerebellum and cortex are implicated in balance control (for a review, see [3]). Disturbances in any of the systems that govern balance may result in balance disorders, e.g., due to reduced vestibular functioning or due to problems with the regulation of tonic motor output. Perhaps surprisingly, balance disturbances can also result from excessive activity in limbic structures that subserve emotionality, in particular fear and anxiety. Several studies have found impaired balance in individuals with anxiety disorders and, conversely, elevated levels of anxiety among individuals with vestibular disorders [4-6]. These patterns of comorbidity suggest that balance disorders and anxiety disorders share a common pathology. As argued in the literature (e.g. [7,8]), this comorbidity is likely mediated by shared neural circuits, in particular the parabrachial nucleus network. The parabrachial nucleus is a major brain stem relay centre for visceral information that includes a vestibulo-recipient region as well as projections to the vestibular nuclei. It has also reciprocal connections with the central amygdaloid nucleus and has been frequently cited as a substrate for anxiety and panic disorders [7].

If there is indeed a link between the neural structures that govern balance and those that govern anxiety, then balance disorders may - in principle - benefit from interventions aimed at reducing anxiety. Conversely, individuals with anxiety disorders should benefit to some extent from balance training. As a case in point, it was recently shown that a program involving 12 weekly sessions involving balance training resulted not only in improved balance, but also in reduced anxiety and higher self-esteem in a group of children with comorbid balance disorders and elevated levels of anxiety [9]. A thorough understanding of the interaction between balance and anxiety in children is especially needed as children continue to develop, and their pathology may start a cycle involving avoidance of balance challenging situations (e.g., on the playground), fewer social and physical encounters, and increased risk of anxiety [10]. But only very few studies have examined postural performance in a group of children with anxiety disorders. It was found [10] that this group of children made more balance mistakes than controls on a wide variety of balance tests, such as walking on a rope. However, no group differences were found in less challenging situations, such as standing heel-to-toe for a certain amount of time. They [10] also found elevated levels of dizziness and sensitivity to motion sickness in their clinical sample, although neurological examination revealed no vestibular impairment. It was concluded that childhood anxiety is characterized by subclinical levels of balance disorder.

Further insight into the interaction between balance and anxiety in children can be gained by using posturographic measures that capture the fine-grained spatio-temporal structure of the naturally occurring body sway during quiet stance. Analysis of the center of pressure (COP) time series can be used to reveal essential properties of the balance system, such as its overall stability, its regularity and complexity, and the attentional involvement in balance regulation, all of which have been considered markers of the quality of postural performance [11-13]. To our knowledge, only two studies have examined postural regulation using a force platform in a child psychiatric population [14,15]. In one study [14] postural regulation in children with Gilles-de-la-Tourette syndrome (TS) was examined. That study found an increase in sway area and an increase in sway velocity in the TS group relative to typically developing children, regardless of whether the eyes of the participants were open or closed during stance. The other study [15] examined children with attention deficit/hyperactivity disorder (ADHD), and here mild postural abnormalities (increased sway area) and mild gait abnormalities were found, regardless of ADHD subtype.

The present research focuses on the interface of childhood anxiety and balance regulation, by means of posturographic measurements. We examined postural performance of a group of children with (sub)clinical anxiety levels under various conditions where balance was challenged. The aim was to reveal which balance parameters related to sway magnitude, sway velocity, and complexity of postural sway would reveal group differences in postural regulation during quiet standing. Our main hypotheses were that the postural sway of high anxious children would (a) have overall greater magnitude (suggestive of lower stability, e.g. [16]), (b) have overall greater velocity (suggestive of greater open-loop control, e.g. [17,18]), and (c) be less complex than the sway of typically developing children. With respect to the latter, there is an emerging view that complexity of physiological time series such as cardiovascular time series [11] and COP fluctuations (e.g. [12]) is indicative of the capacity of the system to adapt to a constantly changing environment (see [19]). Complexity can be thought of as reflecting the information content (entropy) in a time series, and recent studies have shown that postural sway in pathologies such as stroke [12] and cerebral concussion [20] is indeed characterized by lower entropy than that of controls. As argued by some [12,21,22] it could be that reduced entropy reflects the extent to which actors invest attention in their maintenance of posture, which under normal circumstances takes place in a nearly automatic fashion. Based on these considerations we predicted that the reduced postural capabilities of anxious children become manifested as lower complexity in the time series of their posturograms.

In addition, we examined the prediction following from an earlier study [10] that putative group differences become even more apparent when balance is challenged (for comparable findings with a group of children with Developmental Coordination Disorder see [23]). To this end, balance was challenged by increasing the task difficulty in three different ways, namely by removing vision, by having participants stand on a compliant surface (e.g. [24]), and by imposing an attention-demanding cognitive task. Based on findings that adults with increased anxiety levels have greater reliance on visual information for balance [25], we predicted that especially our sample of high anxious children would show excessive sway when no vision was available.

## Methods

### Participants

Eleven children (8 males, 3 females, mean age: 10.3 yr., SD: 1.2, range 8-12) were recruited at Symfora Group Fornhese, a psychiatric unit for child and youth psychiatry in Amersfoort, the Netherlands. The children were referred to this unit by their general practitioner for diagnosis and treatment for various psychiatric problems, possibly related to ADHD or Obsessive Compulsive Disorder (OCD). Inclusion in the present study was based on the outcome score in the borderline or clinical range of the anxiety/depression scale of the Dutch version of the Child Behaviour Checklist (CBCL; [26]; see below) that was administered as part of the standard procedure at admission in the unit. Exclusion criteria were physical limitations that might influence the balance measurements, and an IQ-score below 80. At the time of testing, psychiatric diagnosis was still not fully established. Thirteen typically developing children (4 males, 9 females, mean age: 10.1 yr., SD: 1.3, range 8-12), without known (sub)clinical anxiety levels or psychiatric disorders served as a control group. The study was approved by the local ethics committee before it was conducted.

### Procedure and apparatus

Balance and anxiety measures were completed at Fornhese (anxiety group [AN]) and at the Faculty of Human Movement Sciences (typically developing [TD] group). Parents gave written informed consent and children assented to participate in the study. The CBCL was completed once more by the participants' parents within a few days following the day of testing.

Participants stood barefoot on a 1 × 1 m custom made strain gauge force plate, with their arms hanging relaxed alongside their body. On all trials the same foot placement was adopted (heels 8.4 cm apart, toes pointing outward at an angle of 9 degrees from the sagittal midline). The postural sway of the participants was registered while they performed three different tasks: standing with no additional challenge; baseline (BS), standing on a compliant surface (foam; 40 × 40 × 8 cm, medium density) (Foam Standing; FS), and standing while performing a cognitive dual task (DT). All conditions were performed with eyes open (EO) and eyes closed (EC), giving rise to six conditions. Each of these conditions was repeated 5 times, resulting in a total of 30 trials per participant, presented in fully randomized order. Between each block of 6 trials a small break was given, during which participants were able to freely move and walk around. Participants were instructed to maintain quiet stance during the measurements. During eyes open trials, participants were instructed to focus on a drawing located at eye level, 1.5 m in front of them. The dual task consisted of a memory task. During these trials, participants had to listen to a list of animal names. The words were presented at a frequency of 0.5 Hz, which resulted in a total number of twelve different animal names per trial. Participants were instructed to fully concentrate on the names and to memorize as many of the names as they could. After completion of the DT trial, participants verbally reported the animal names they remembered. The number of correctly remembered items was scored by the experimenter.

COP data were collected for 20 s at a sample frequency of 200 Hz. The data collection started after the participant stood still for five seconds. An experimenter stood behind the participant during all trials for safety reasons.

### Anxiety measures

In order to assess the level of anxiety two different measures were used. The level of experienced (state) anxiety of the participant was examined by asking participants to scale their current anxiety level immediately prior to the experiment on an anxiety thermometer. The anxiety thermometer runs from 0 to 10, with 0 corresponding to 'no anxiety' and 10 to 'extremely frightened' [27].

In addition, we assessed trait anxiety a few days following testing based on the scores of the Dutch version of the Child Behaviour Check List. The CBCL is a parent-rating scale to assess various aspects of behavior and psychopathology in childhood. The test-retest reliability of the CBCL and the internal consistency of the scales are both good (for details see [26]). The CBCL consists of two scales; a social competence scale and a behavior problem scale. Only the behavior problem scale was used for our purposes. The behavior problem scale consists of 113 items describing possible behaviors that the child may or may not exhibit. The items are grouped in eight different syndrome scales, and we focused on the scores of the anxious/depressed sub-scale. The calculated scores of each domain can be classified as 'normal ' (T-scores ≤ 59), 'borderline' (60 ≤ T-scores ≤ 63), or 'clinical range' (T-scores ≥ 64). Scores in the borderline range are often considered high enough to be of concern.

### Posturographic data analysis

The continuous displacement of the COP was calculated in *x *(medio-lateral [ML]) and *y *(anterior-posterior [AP]) directions. Prior to all analyses the mean was subtracted from both medio-lateral and anterior-posterior COP trajectories to correct for offset. The posturographic time series were bi-directionally filtered (2^nd ^order low pass Butterworth filter, cut-off frequency of 12.5 Hz). In addition the radial component, or resultant distance (*r*), was calculated following [13], with *i *= 1, 2, 3, ..., *N *and *N *indicating the total number of data points in the COP time series (i.e., 3999).

The amount of postural sway was quantified by means of the sway area (SA), a statistically based estimate of a confidence ellipse that encloses approximately 95% of the points of the COP trajectory. The ellipse was calculated using the following equation:



where *F*._05 [2, *N*-2] _is the *F *statistic at a 95% confidence level for a bivariate distribution with *N *data points. If *N *is > 120, *F *is 3.00. *s*_AP _and *s*_ML _are the standard deviations of the AP and ML time series respectively, while *s*_APML _represents their covariance [13].

Average sway velocity was determined by calculating the sum of the COP displacements in the AP-ML plane over a trial (i.e., the sway path length) and dividing this number by the recording time, i.e., 60 s.

To examine the structure of the COP trajectories independent of its size or scale, *x *and *y *were normalized to unit variance by dividing the time series by their respective standard deviations. The sway path length calculated over this normalized posturogram provides a scale free measure of the amount of 'twisting and turning', in which larger SP_*n *_values indicate more twisting and turning [22]. This measure is thus related to the spatial complexity of the COP time series:



Finally, to gain insight into the complexity of the time series we calculated the sample entropy. The sample entropy (SampEn) in a set of data points is the negative natural logarithm of the conditional probability that a sequence of data points with length *N*, having repeated itself within a tolerance *t *for *M *points, will also repeat itself for *M *+ 1 points, without allowing self-matches [11]. SampEn provides information about the regularity of a time-series, whereby a decrease in SampEn values implies an increase in regularity. Low regularity has been associated with a more flexible and healthy pattern, as healthy physiological systems (e.g., the human heart) are often characterized by an irregular and complex type of variability [11,19] whereas in the presence of pathology or aging more regular (and thus less complex) behavior can be observed [12,21,22,28]. SampEn was calculated on the radial COP components, normalized to unit variance. SampEn software was obtained from PhysioNet. Parameter values of *M *(*M *= 3) and *t *(*t *= 0.05σ) were based on earlier studies [12,29] to find optimum values for these parameters.

### Statistical Analysis

The posturographic data of all dependent measures were averaged over the five trials of each condition. A repeated measures analysis of variance (ANOVA) was used with within-subject factors task (3 levels: BS, FS, and DT) and vision (2 levels: EO and EC), and group (anxiety [AN] and control, i.e. typical development [TD]) as the between-subject factor on postural sway parameters. Possible interactions were explored using follow-up analyses.

Independent-samples *t*-tests were performed to test for differences between the experimental and the control group on (a) the anxiety thermometer scores, (b) scores of the anxiety/depression scale of the CBCL, and (c) performance on the memory task (number of correctly recalled items). For all analyses we adopted a *p*-value of .05.

## Results

### Data evaluation

The data of two participants, both from the TD group, had to be excluded from the study; the posturographic data of one child showed unexplainable artefacts, while the other child showed elevated levels of anxiety on the anxiety measures. Furthermore, three AN children lost their stability on one occasion in the foam condition. These trials were excluded from the posturographic analyses.

### Anxiety measures

Statistical analyses of the CBCL did show a significant difference between the anxiety and control group on the 'anxious/depressed' scale, *t*(20) = 5.701, *p *< .001, whereby the anxiety group scored in the clinical range (mean 65.2; SD 5.9), whereas all children in the TD group scored in the normal range (mean 52.2; SD 4.4). There were no significant differences between the anxiety levels of groups on the anxiety thermometer (*t*(20) = 0.539, *p *> .1). As the groups did not differ in experienced state anxiety before the onset of the experiment, possible posturographic differences may therefore be due to differences in stable subject characteristics.

### Memory performance

Children in the AN group recalled significantly fewer items than children in the TD group, *t*(20) = 2.920, *p *< .01 (mean 4.2 vs. 5.8 items, respectively).

### Posturographic measures

#### Amount of sway

Main effects of group, *F*(1, 20) = 16.847, *p *< .001, task, *F*(2, 40) = 45.850, *p *< .001, and vision, *F*(1, 20) = 17.064, *p *< .001, were found. The main effect of group indicated that sway area was overall larger for the anxiety group (AN) than the typical developmental group (TD). The main effect of task was due to significantly elevated levels of postural sway in the foam condition, compared to the baseline and cognitive dual task condition. Also, removal of vision led to increased postural sway.

In addition to these main effects significant two-way interactions were found, Task × Vision, *F*(2, 40) = 43.373, *p *< .001, and Task × Group, *F*(2, 40) = 7.590, *p *< .001. The first can be explained by the fact that removal of vision resulted in a larger sway area, but mainly when participants stood on foam. The second revealed that both groups responded differently to the task manipulations; the anxiety group exhibited greatly elevated levels of sway when standing on foam.

**Figure 1:**
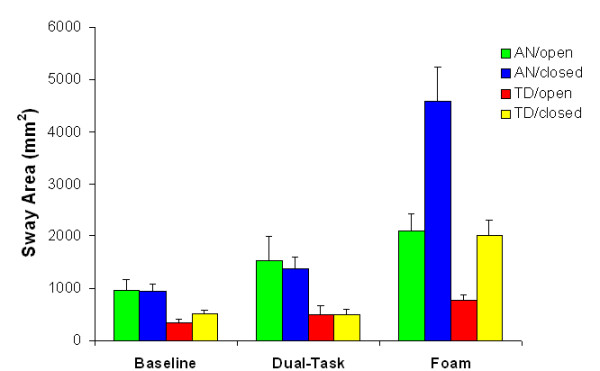
**Sway Area (mean + s.e.m.) as a function of group, vision and task**. AN = anxiety group, TD = typical developing group.

Finally, these two-way interactions were modulated by a significant Task × Vision × Group interaction, *F*(2, 40) = 6.124, *p *< .001. As can be seen in Figure 1 the sway area was much larger during the condition where AN participants stood with their eyes closed on the foam surface than during all other conditions. The interaction was explored by performing separate 2 × 2 ANOVA's for each task (BS, FS and DT), with group and vision as factors. As expected, the Group × Vision interaction was only significant for the FS task, *F*(1, 20) = 4.310, *p *< .05, and not for the other tasks. It thus seems to be the case that the three-way interaction was caused by extreme values in one particular condition, namely the condition where AN children maintained balance under the most challenging circumstances (foam, eyes closed).

#### Sway velocity

We found main effects of group, *F*(1, 20) = 7.601, *p *< .05, task, *F*(2, 40) = 73.063, *p *< .001, and vision, *F*(1, 20) = 69.513, *p *< .001. The main effect of group indicated that sway had overall greater velocity for the anxiety group (AN) than the typical developmental group (TD). The main effect of task was due to significantly elevated levels of sway velocity in the foam condition, compared to other conditions. Also, removal of vision led to higher sway velocity.

We found two two-way interactions, Task × Vision, *F*(2, 40) = 112.230, *p *< .001, and Task × Group, *F*(2, 40) = 3.316, *p *< .05. The first can be explained by the fact that standing with eyes closed on the foam surface resulted in faster body sway than the other conditions. The second was due to high sway velocity for the anxiety group when standing on foam.

**Figure 2:**
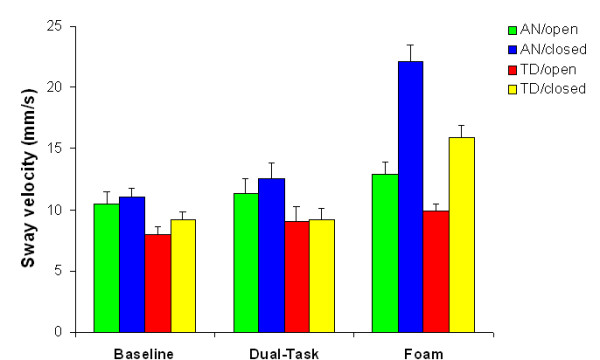
**Sway velocity (mean + s.e.m.) as a function of group, vision and task**. AN = anxiety group, TD = typical developing group.

Finally, the three-way Task × Vision × Group interaction was significant *F*(2, 40) = 6.191, *p *< .001. Similar to the findings with the sway area, this was due to rather high sway velocity values in one particular condition, namely the condition where AN children maintained balance under the most challenging circumstances (foam, eyes closed). Mean values across groups and condition are shown in Figure 2.

#### Normalized sway path length

The main effect of group, *F*(1, 20) = 7.549, *p *< .05, on the normalized sway path length (SPn) showed that the AN group exhibited a significantly overall shorter sway path than the TD group. In other words, the anxiety group showed less 'twisting and turning' during quiet standing than the control group. The main effect of task, *F*(1,20) = 18.715, *p *< .001, revealed that SPn values were significantly lower in the foam condition than in the other two conditions (BS-DT: *t*(21) = 0.474, *p *> .1; BS-FS: *t*(21) = 3.333, *p *< .05; FS-DT: *t*(21) = 3.562, *p *< .05).

**Figure 3:**
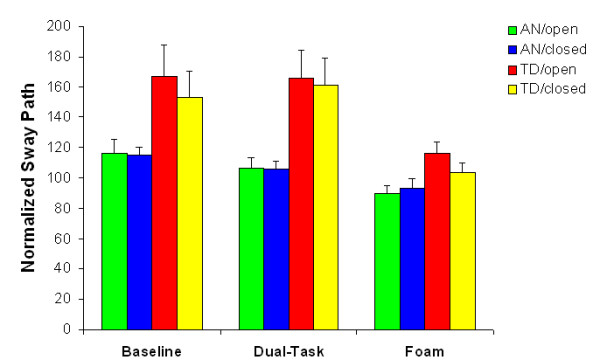
**Normalized Sway Path length (mean + s.e.m.) as a function of group, vision and task**. AN = anxiety group, TD = typical developing group.

Both main effects were modulated by a significant Task × Group interaction, *F*(2, 40) = 4.047, *p *< .05. As can be seen in Figure 3, there was a larger decrease in SPn values for the TD group than for the AN group when standing on foam.

#### Sample Entropy

**Figure 4:**
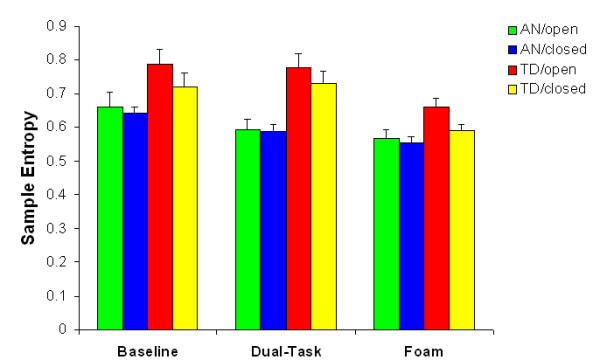
**Sample Entropy (mean + s.e.m.) as a function of group, vision and task**. AN = anxiety group, TD = typical developing group.

Statistical analysis of the SampEn values revealed main effects of group, *F*(1, 20) = 9.667, *p *< .05, task, *F*(1, 20) = 16.841, *p *< .001, and vision, *F*(1, 20) = 10.594, *p *< .05. The sway path of the AN group exhibited lower SampEn values than the control group, indicating greater regularity of the COP time series. The effect of task was due to reduced SampEn values during standing on foam relative to the other two conditions. Finally, removal of vision led to a decrease in SampEn compared to when the eyes were open (see Figure 4).

In addition, a significant Task × Group interaction was found, *F*(2, 40) = 3.245, *p *< .05, which can be explained by the fact that there was only a significant difference in SampEn between the groups during the more challenging tasks (FS: *t*(21) = 2.445, *p *< .05, DT: *t*(21) = 3.778, *p *< .001) and not during normal standing (BS; *t*(21) = 1.956, *p *> .1), regardless of whether vision was available. There was also a significant Vision × Group interaction, *F*(2, 40) = 4.618, *p *< .05. Post hoc analyses revealed that the main effect of vision only applied to the TD group: removal of vision induced significantly lower SampEn values for this group, *t*(10) = 4.735, *p *< .001, whereas removal of vision did not lead to a change in regularity for the AN group (*t*(10) = 0.673, *p *> .1).

## Discussion

The aim of the present study was to gain further insight into the balance-anxiety link, using posturographic measures. We examined the behavior of the naturally occurring body sway in a group of children with elevated anxiety levels and an age-matched control group, using a variety of sensory and cognitive manipulations. The analyses focused on theoretically motivated measures related to postural stability and the complexity of the COP time series. The main results can be summarized as follows.

First, as hypothesized we found evidence for the presence of sub-clinical postural anomalies in children with elevated levels of anxiety. The COP fluctuations of the anxiety group during normal standing exhibited a larger sway area than the controls. This is suggestive of lower postural stability, although it should be noted that the precise relation between the amount of sway and stability of the human "inverted pendulum" remains to be elucidated (e.g. [30]). Also, COP movements were relatively fast in the anxiety group. It has been argued that high sway velocity is indicative of greater open-loop regulation of balance [18], as opposed to closed-loop (automatized) balance, which could mean that the anxiety group is less reliant on automatized postural control processes. In addition, we found that the COP fluctuations of the anxiety group were on average less complex than those of controls, as exemplified by shorter normalized sway path (suggestive of fewer corrective sub-movements) and greater regularity, which is also in line with our hypothesis. These latter two measures have been linked to the amount of attention invested in the regulation of balance. As argued in the Introduction, postural sway of patients with neurological disorders such as stroke or concussion is characterized by greater regularity, which is suggestive of increased cognitive control to compensate for the reduced capabilities of the motor system to operate in an automatic and fluent fashion. In relation to this, it was found [12] that regularity of the COP fluctuations of stroke patients decreased again in the course of rehabilitation, which was interpreted as a progressive reduction in attentional investment, i.e., increased automaticity of postural regulation. Another study [31] examined the other end of the hypothesized automaticity continuum, and it was found that the COP of young skilled dancers was even less regular than that of healthy controls, suggesting even less cognitive investment in balance, i.e., a more fully automatized form of balance control. Thus, the present findings suggest that children with elevated levels of anxiety utilize excessive attentional resources for the maintenance of posture. Under normal circumstances the regulation of balance takes place in a (nearly) fully automatic manner, which leaves the actor enough room to allocate attention to other tasks, such as talking, thinking, or visual search. Our results imply that children with elevated levels of anxiety will be less capable of dividing attention between the regulation of balance and cognitive secondary tasks. Future studies will have to control for differences in cognitive capability, in order to test this hypothesis more rigorously.

Second, we found that the availability of visual information affected the COP. When standing with eyes closed there was an overall increase in body sway an increase in sway velocity, and an increase in the regularity of the COP fluctuations, compared to quiet standing with the eyes open. Although it is usually assumed that increase in body sway with eyes closed is due to loss of stability as a result of the removal of a crucial source of information for the regulation of balance, it could also be the case that the increase in sway reflects that actor's attempt to increase the reliability of vestibular and proprioceptive channels. From this perspective, actors can make a conscious effort to compensate for their loss of vision by increasing the amount of self-generated exploratory motor activity, in the service of facilitating or sensitizing alternate sources of information. Although we found no interaction between group and vision on the amount of body sway, this interaction was significant for the regularity of sway. We found that closing the eyes led to more regularity (suggestive of greater attentional involvement in balance regulation), but only for the TD group, which was contrary to our expectations. It could be the case that children in the TD group changed their postural strategy from automatic control (eyes open) to a strategy involving attentional control (eyes closed), whereas the children in the anxiety group used attentional regulation of balance throughout the experiment, that is, regardless of the availability of visual information.

Third, we found that postural sway was hardly affected by the cognitive secondary task (DT). The only reliable finding involving cognition was a decrease in SampEn for the AN group, suggesting that performing a challenging secondary task (word memorization) led to an even greater attentional involvement in balance for the anxiety group, relative to controls. Although effects of cognition on postural fluctuations are commonly found, the literature is actually quite inconsistent, and at present no firm conclusions can be drawn regarding the way concurrent cognitive tasks impact on the regulation of balance [30].

Fourth, we found that balance was strongly affected by standing on a compliant surface. When standing on foam there was an increase in body sway, an increase in sway velocity, a decrease in the length of the normalized sway path, and an increase in the regularity of the COP fluctuations, compared to standing on a rigid surface. Note that standing on foam reduces the reliability of proprioceptive information from the ankles. This, in turn, may again lead to reduced postural stability, and a greater need to invest attentional resources in the maintenance of balance, resulting in lower complexity of the signal. Importantly, the anxiety group reacted strongly to this manipulation, especially with eyes closed. There was a 4- to 5-fold increase in the size of the sway area in the AN group when standing on foam with eyes closed, compared to standing on a steady surface with eyes open (see Figure 1). The results also clearly show that when the balance task became more difficult the differences in postural performance between the subject groups became greater. This is fully in line with the study described earlier [10], where it was found that maintaining balance on an unsteady surface (a trampoline) resulted in a disproportionate increase in balance mistakes in the group of children with anxiety disorder, compared to controls.

At present, only very few studies have examined postural behavior in various psychopathologies. Sub-clinical postural anomalies were found in children with Tourette syndrome [14], adult obsessive-compulsive disorder patients [32], and children with ADHD [15]. In all these studies it was theorized that neural structures involved in psychopathology and structures involved in balance regulation share a common network. More specifically, some authors [14] speculated that the observed postural anomalies were due to impaired feedback processing, associated with fronto-striatal dysfunction. Along similar lines, others [32] proposed that prosencephalic structures - involved in anxiety - can influence the vestibular system via the parabrachial nucleus. It has also been suggested that postural dysregulation could be related to mild cerebellar dysfunction [15]. At present, it is unknown whether psychopathology can directly dysregulate the neural systems that subserve the regulation of balance, or whether some unknown brain lesion (either hereditary or acquired) simultaneously affects a number of subsystems in the course of brain maturation, which would be consistent with the notion of 'atypical brain development' (e.g. [33]). According to this notion, developmental brain disorders can result in a spectrum of seemingly unrelated disabilities, resulting in unique neuropsychological profiles that do not fit nicely in pre-existing diagnostic categories. Comorbidity (or "co-occurrence" [34]) of neuropsychiatric problems is therefore the rule, not the exception. The studies cited above all reported comorbid disorders in their sample, and they acknowledged that the heterogeneity of the patients groups precludes drawing strong conclusions. With respect to our clinical sample, the children had behavior problems related to ADHD and OCD, so that our observation of postural anomalies cannot be attributed exclusively to anxiety. Future studies will have to reduce the within group variance by either using more heterogeneous samples (preferably also gender-matched), or through appropriate statistical techniques such as multiple regression. In addition, brain imaging studies can shed valuable light on which brain structures (limbic, motor, or otherwise) are affected.

## Limitations

A clear limitation concerns the heterogeneity of the sample in terms of clinical status. Relatedly, possible comorbidity with other disorders puts a limit on the generalizability of the findings. However, the findings are consistent with the emerging view that anxiety disorders and balance performance are intertwined.

## Conclusion

We found postural anomalies in children with elevated anxiety levels. The children exhibited overall more regular postural sway even for the simplest balance task which suggests that the underlying postural control is qualitatively different from children without elevated anxiety. We postulated that these anomalies are in part due to an excessive attentional focus (possibly related to hypervigilance) to the own body. The present study is consistent with the increasing awareness in the psychiatric field that neurodevelopmental disorders may benefit from body-oriented treatment approaches.

## Competing interests

The authors declare that they have no competing interests.

## Authors' contributions

JS analyzed the data and drafted the manuscript. AL and CE coordinated the study and commented on the written drafts of the manuscript including interpretation of the results. EHVD collected the data, performed the data analysis and was involved in drafting the manuscript. PJB was involved in drafting and revising the manuscript. All authors read and approved the final manuscript.

## References

[B1] Horlings CGC, Küng UM, Bloem BR, Honegger F, van Alfen N, van Engelen BGM, Allum JHJ (2008). Identifying deficits in balance control following vestibular or proprioceptive loss using posturographic analysis of stance tasks. Clin Neurophysiol.

[B2] Loram ID, Maganaris CN, Lakie M (2005). Active, non-spring-like muscle movements in human postural sway: how might paradoxical changes in muscle length be produced?. J Physiol.

[B3] Lalonde R, Strazielle C (2007). Brain regions and genes affecting postural control. Prog Neurobiol.

[B4] Balaban CD, Jacob RG (2001). Background and history of the interface between anxiety and vertigo. J Anxiety Disord.

[B5] Kogan E, Lidor R, Bart O, Bar-Haim Y, Mintz M (2008). Comorbidity between balance and anxiety disorders: verification in a normal population. J Psychol.

[B6] Sklare DA, Konrad HR, Maser JD, Jacob RG (2001). Special issue on the interface of balance disorders and anxiety. An introduction and overview. J Anxiety Disord.

[B7] Balaban CD (2002). Neural substrates linking balance control and anxiety. Physiol Behav.

[B8] Balaban CD, Thayer JF (2001). Neurological bases for balance-anxiety links. J Anxiety Disord.

[B9] Bart O, Bar-Haim Y, Weizman E, Levin M, Sadeh A, Mintz M (2009). Balance treatment ameliorates anxiety and increases self-esteem in children with comorbid anxiety and balance disorder. Res Dev Disabilities.

[B10] Erez O, Gordon CR, Sever J, Sadeh A, Mintz M (2004). Balance dysfunction in childhood anxiety: findings and theoretical approach. J Anxiety Disord.

[B11] Richman JS, Moorman JR (2000). Physiological time-series analysis using approximate entropy and sample entropy. Am J Physiol Heart Circ Physiol.

[B12] Roerdink M, de Haart M, Daffertshofer A, Donker SF, Geurts ACH, Beek PJ (2006). Dynamical structure of center-of-pressure trajectories in patients recovering from stroke. Exp Brain Res.

[B13] Prieto TE, Myklebust JB, Hoffmann RG, Lovett EG, Myklebust BM (1996). Measures of postural steadiness: differences between healthy young and elderly adults. IEEE Trans Biomed Eng.

[B14] Lemay M, Termoz N, Lesperance P, Chouinard S, Rouleau GA, Richer F (2007). Postural control anomalies in children with Tourette syndrome. Exp Brain Res.

[B15] Buderath P, Gärtner K, Frings M, Christiansen H, Schoch B, Konczak J, Gizewski ER, Hebebrand J, Timmann D (2009). Postural and gait performance in children with attention deficit/hyperactivity disorder. Gait Posture.

[B16] Schmit JM, Regis DI, Riley MA (2005). Dynamic patterns of postural sway in ballet dancers and track athletes. Exp Brain Res.

[B17] Deconinck FJA, De Clercq D, Van Coster R, Oostra A, Dewitte G, Savelsbergh GJP, Cambier D, Lenoir M (2007). Sensory contributions to balance in boys with developmental coordination disorder. Adapt Phys Activ Q.

[B18] Riach CL, Starkes JL (1994). Velocity of centre of pressure excursions as an indicator of postural control systems in children. Gait Posture.

[B19] Duarte M, Sternad D (2008). Complexity of human postural control in young and older adults during prolonged standing. Exp Brain Res.

[B20] Cavanaugh JT, Guskiewicz KM, Giuliani C, Marshall S, Mercer VS, Stergiou N (2006). Recovery of postural control after cerebral concussion: new insights using approximate entropy. J Athl Train.

[B21] Donker SF, Ledebt A, Roerdink M, Savelsbergh GJP, Beek PJ (2008). Children with cerebral palsy exhibit greater and more regular postural sway than typically developing children. Exp Brain Res.

[B22] Donker SF, Roerdink M, Greven AJ, Beek PJ (2007). Regularity of center-of-pressure trajectories depends on the amount of attention invested in postural control. Exp Brain Res.

[B23] Geuze RH (2003). Static balance and developmental coordination disorder. Hum Mov Sci.

[B24] Patel M, Fransson PA, Lush D, Gomez S (2008). The effect of foam surface properties on postural stability assessment while standing. Gait Posture.

[B25] Redfern MS, Furman JM, Jacob RG (2007). Visually induced postural sway in anxiety disorders. J Anxiety Disord.

[B26] Achenbach TM, Rescorla LA (2001). Manual for the ASEBA School-Age Forms & Profiles.

[B27] Houtman ILD, Bakker FC (1989). The anxiety thermometer: a validation study. J Pers Assess.

[B28] Pincus SM, Goldberger AL (1994). Physiological time-series analysis: what does regularity quantify?. Am J Physiol Heart Circ Physiol.

[B29] Lake DE, Richman JS, Griffin MP, Moorman JR (2002). Sample entropy analysis of neonatal heart rate variability. Am J Physiol: Regul Integr Comp Physiol.

[B30] Fraizer EV, Mitra S (2008). Methodological and interpretive issues in posture-cognition dual-tasking in upright stance. Gait Posture.

[B31] Stins JF, Michielsen ME, Roerdink M, Beek PJ (2009). Sway regularity reflects attentional involvement in postural control: effects of expertise, vision and cognition. Gait Posture.

[B32] Kemoun G, Carette P, Watelain E, Floirat N (2008). Thymocognitive input and postural regulation: a study on obsessive-compulsive disorder patients. Neurophysiol Clin.

[B33] Gilger JW, Kaplan BJ (2001). Atypical brain development: a conceptual framework for understanding developmental learning disabilities. Dev Neuropsychol.

[B34] Kaplan BJ, Dewey DM, Crawford SG, Wilson BN (2001). The term comorbidity is of questionable value in reference to developmental disorders: Data and theory. J Learn Disabil.

